# Introduction to the Special Issue on *Clostridioides difficile*

**DOI:** 10.3390/antibiotics10101233

**Published:** 2021-10-11

**Authors:** Guido Granata, Davide Roberto Donno

**Affiliations:** Clinical and Research Department for Infectious Diseases, National Institute for Infectious Diseases L. Spallanzani, IRCCS, 00149 Rome, Italy; davideroberto.donno@inmi.it

The Gram-positive, anaerobic bacterium *Clostridioides difficile* (CD) represents the most common cause of nosocomial diarrhea worldwide and is responsible for increased morbidity and mortality, and prolonged hospital stays [1,2]. Indeed, despite the large number of scientific publications exploring the epidemiology and the clinical management of *Clostridioides difficile* infection (CDI), there is still a huge need for studies that could clarify some important aspects of this complex disease.

First, the clinical spectrum of CDI varies in severity from asymptomatic carriage and self-limited, mild diarrhea to severe colitis, toxic megacolon, and death [3]. Mortality rates in CDI vary widely between studies, from less than 2% up to 17% [4,5,6]. Besides the well-known risk factors for CDI, there is a need for tools to early identify CDI patients, particularly patients at high risk for severe CDI, and recurrence of CDI [4,7].

Another issue is hospital spreading of CD. Understanding the routes of in-hospital and community CD transmission is crucial to develop specific interventions to reduce the spread of CDI. The definition and validation of antimicrobial stewardship programs on CD prevention may reduce CDI incidence, even during the COVID-19 pandemic [8].

A further uncertainty is placed on the molecular pathogenesis of CDI, including a more exhaustive description of the interplay between CD, the gut microbiota, and host immunity, as well as the exact role of CD toxins, i.e., toxin A, toxin B, and binary toxin. 

Finally, no less important is the high recurrence rate observed with the currently available CDI therapy. Recurrences currently represent one of the major challenges in the management of CDI, resulting in higher hospitalization costs and in increased morbidity and mortality rates [3,4,9]. The currently recommended first-line antimicrobial therapy is represented by oral vancomycin or fidaxomicin for the first episode. Recently, new innovative approaches, based on non-antimicrobial compounds, i.e., monoclonal anti-toxin antibodies, fecal microbiota transplantation, live bacterial vaccines, and CD vaccines, have been developed. However, further studies are needed to confirm the efficacy and safety of these approaches.

This Special Issue includes ten full research articles and one review article. These contributions shed rays of light on several gray areas of our knowledge of CDI, including regional CD phenotypic and genotypic patterns, the CDI clinical course and the mortality rate in different settings and patient case-mix, the levels of CD toxin A and toxin B in the serum of CDI patients, and several novel therapeutic approaches.

The report by Wongkuna et al. showed data addressing ribotypes, toxigenicity, and antimicrobial susceptibility profiles of two series of CD isolates in Thailand [10]. The authors compared two series of CD isolates, including 50 isolates collected from 2006 to 2009 and 26 isolates collected from 2010 to 2012. Interestingly, as ribotype 017 was the most common in both groups, 18 ribotyping patterns previously unknown were identified in the region. This work provides evidence of temporal changes in CD strains in Thailand [10].

The contribution by Novakova et al. reported the local phenotypic (toxigenicity, antimicrobial susceptibility) and genotypic (PCR ribotypes, genes for binary toxins) patterns of CD isolates from CDI patients hospitalized in the region of northern Slovakia, reporting a high prevalence of CD ribotype 176 and ribotype 001 [11]. 

Regarding CDI pathogenesis, the work by Di Masi et al. presented a novel semi-quantitative diagnostic method to measure the serum levels of CD toxins, i.e., toxin A and toxin B [12]. By the use of this new assay, the authors report the detection of toxemia in 33 out of the 35 CDI cases included in the study. The relationship between toxin A serum levels and CDI severity was also assessed, reporting that, at the time of CDI diagnosis, the proportion of severe CDI cases with a toxin A serum level >60 pg/µL was higher than in mild CDI cases (29.4% versus 66.6%, *p* = 0.04) [12]. Of interest, this study showed that toxemia is much more frequent than expected in CDI patients, and that high serum levels of toxin A correlate with CDI severity [12].

Among the contributions included in this Special Issue, a large multicenter study was performed by members of the European Society of Clinical Microbiology and Infectious Diseases (ESCMID) Study Group for CD [13]. This retrospective, case–control study aimed to describe the risk factors, clinical presentation, and management of patients with CDI as well as reporting factors associated with mortality in the 90-day period after diagnosis. In this study, 415 CDI patients hospitalized between January 2011 and December 2019 who died within 90 days following a CDI diagnosis formed the case group that was compared in a 2:1 ratio to 209 control CDI patients hospitalized in the same wards over the same time period who survived. The study found that older age, inadequate CDI therapy, cachexia, malignancy, Charlson index, long-term facility care, elevated white blood cells, elevated C-reactive protein, bacteremia, and cognitive impairment were independent risk factors for mortality at day 90 [13]. Notably, the authors concluded that CDI prevention should be primarily focused on hospitalized elderly people receiving antibiotics. For these patients, the available preventive measures should be used all the time, instead of only after CDI diagnosis.

Regarding the risk for the development of CDI, patients with liver dysfunction, including nonalcoholic fatty liver disease patients and cirrhotic patients, deserve attention. Cirrhotic patients are vulnerable to developing CDI due to their frequent admission and infections, as well as dysbiosis and a low immune system. Considering this, variceal bleeding secondary to cirrhosis requires antibiotics to prevent bacterial translocation, and thus patients become susceptible to CDI. The study by Voicu et al. aimed to investigate the risk factors for CDI in cirrhotic patients with variceal bleeding and the mortality risk in this patient population [14]. This retrospective cohort study included 367 cirrhotic patients with variceal bleeding, from which 25 patients were confirmed to have CDI. The authors reported that a higher age, longer hospital stay, higher level of urea, higher Charlson index, and the use of proton pump inhibitors were risk factors for CDI in cirrhotic patients [14]. Moreover, the authors confirmed that the MELD score was a predictor for mortality in cirrhotic patients with CDI. Moreover, the authors proposed a model of four predictors (age, days of admission, Charlson index, Child–Pugh score) to assess the risk of CDI in cirrhotic patients. Of note, in this study, cirrhotic patients with CDI had significantly higher costs compared with those without CDI [14].

The study by Šamadan et al. evaluated patients with nonalcoholic fatty liver disease and CDI, with the aim to determine whether nonalcoholic fatty liver disease is an independent risk factor associated with CDI recurrence [15]. This retrospective cohort study included 329 hospitalized CDI patients. Of the 329 patients included, 107 patients (32.5%) experienced recurrence of CDI. The statistical analysis identified that Charlson age–comorbidity index >6, age >75 years, nonalcoholic fatty liver disease, chronic kidney disease, and immobility were risk factors associated with recurrence of CDI [15]. Therefore, the authors identified nonalcoholic fatty liver disease as a possible new host-related risk factor associated with recurrence of CDI and suggested that changes in the intestinal microbiota linked to the development and progression of nonalcoholic fatty liver disease are a possible explanation of the increased risk of CDI in these patients.

Regarding the therapeutic approaches to CDI, a contribution by Ojha et al. highlighted that teicoplanin, an antimicrobial agent approved for the treatment of other bacterial infections, prevents the outgrowth of CD vegetative cells [16].

Intriguingly, the manuscript by Heidebrecht et al. proposed, again, the idea to use specific polyclonal antibodies isolated from the milk of immunized cows to treat CDI, in contrast to the standard administration of antibiotics [17]. In this study, the authors focused on the role of the microbiome, collecting stool samples of hamsters with CDI treated with either bovine antibodies or vancomycin. The regeneration of the microbiome instantly begins with the start of the antibody treatment, in contrast to the vancomycin-treated mice, where the diversity decreased significantly during the treatment duration. Of importance, the authors underlined that the regeneration of the microbiome was not an antibody-induced regeneration, but a natural regeneration that occurred because no microbiota-inactivating substances were administered [17]. 

This Special Issue also includes the trial by Pellissery et al. to investigate the prophylactic and therapeutic efficacies of baicalin, a plant-derived flavone glycoside, in reducing the severity of CDI [18]. In the prophylactic trial, mice were provided with baicalin from 12 days before CD challenge through the end of the experiment, whereas baicalin administration started on day 1 post-challenge in the therapeutic trial. The authors reported that both prophylactic and therapeutic supplementation of baicalin significantly reduced the severity of colonic lesions and improved CDI clinical progression and outcome compared with the control. Moreover, the authors highlighted that baicalin supplementation favorably altered the mice microbiome composition [18].

Another innovative anti-CDI compound was described in the contribution by Mahadari et al. [19]. This research group synthesized a compound with acceptable water solubility and a CD-selective antibacterial activity. Promisingly, this novel compound exhibited mild efficacy in an in vivo murine model of CDI, reducing the severity and slowing the onset of disease [19]. 

Finally, in a review article, Shieh and coworkers described the current state of our knowledge about the molecular pathogenesis of CDI and the perspectives of the application of nanotechnologies for the management of CDI, including advantages and limitations. This review highlighted the great potential of nanomedicine as a novel strategy in the future management of CDI [20].

This Special Issue collects multidisciplinary research focused on CDI. The contributions here collected constitute a valuable knowledge reservoir for scientists working in this field. The Guest Editor thanks the scientific community who showed interest in and submitted manuscripts to this collection.
